# Notes on Myotomy and Tenotomy

**Published:** 1841-10-23

**Authors:** Reynell Coates


### NOTES ON MYOTOMY AND TENOTOMY.

BY REYNELL COATES, M. D.

To the Editors of the Medical Examiner.

GENTLEMEN,—If we suppose the mechanical treatment of varus to have been carried out upon the plan or on the principles sketched in the preceding papers of this series, (and it appears plainly to me that, when successfully managed, it must be, in the very nature of things, conducted—intentionally or unintentionally—completely or partially—adroitly or clumsily—on such principles alone,) the resistance of the contracted tendo Achilles is rather an advantage than a disadvantage, until the third and second stages of varus are reduced to the mildest form of the first stage. The forces and circumstances that oppose this reduction, are the contraction of the adductors in part, but far more considerably the shortening of the fascia plantaris and the plantar muscles, with the curtailment in length of many of the tarsal ligaments, and the formation of new ones. That these are all conquerable by mechanical treatment within a very reasonable time, even when that treatment is pursued under the very serious mechanical disadvantages resulting from the division of the tendo Achilles, is as clearly proved by the evidence of the tenotomists themselves, as is any other observation recorded by them. It is folly, then, to contend that the most customary operations of the tenotomists are necessary in removing the peculiar deformities of the second and third stages of varus. It is only in the less common operations—those seldom performed, except by their inventors and a few special disciples,—such as the division of the fascia plantaris recommended by Mr. Coates, that we really facilitate—perhaps at the expense of greater evils—the reduction of such deformities.

The true subject of discussion, then, between the really well informed advocates of the purely mechanical treatment, or, to speak more correctly, *the treatment exclusively by machines*, on the one part, and the defenders of the mixed plan of proceeding by means of *the knife followed by the application of machines*, on the other, is narrowed to this simple question: Is the accomplishment of a perfect cure of pes equinus, or of that slight degree of varus which most resembles it and is often confused with it, accomplished more readily after than before the division of the tendo Achilles?

If we exclude from our calculation a certain malposition of the os calcis, observed in most pedes equini and all cases of true varus of the first degree, the only indication to be fulfilled is, the flexion of the whole foot by means of the permanent elongation of the extensor muscles or their tendon, either by a diminution of their tonic contraction, or a modification of nutrition by means of which their fibres are positively lengthened, or by tenotomy.

All the tenotomists—even the most moderate of the school; Detmold, for instance,—seem, by the phraseology of their directions on this subject, to regard the elongation by diminution of tone, as all or nearly all that can be accomplished by mechanical flexion alone! (see the passage quoted from Detmold, page 598.) This is certainly singular, and will appear unreasonable at once to any surgeon who reflects that merely causing the muscle to "yield to a certain point," when, "on this point being exceeded, it *will react against the extension*," cannot possibly produce any *permanent* benefit! A muscle that has only yielded to extension till it reacts, *and even till it reacts obstinately* against the force employed, will again contract with promptitude as soon as the force employed is employed no longer. Apply this obvious principle in criticising apparatus for the treatment of pes equinus, and it is evident that machinery can only be regarded as rational in its construction, when it is so contrived as to keep up moderate and *endurable* extension of the flexors, not merely until they are exhausted and their tenacity diminished, but until a change of nutrition has actually lengthened their fibres.

Every surgeon well knows that, in any ordinary luxation, certain muscles are violently extended beyond their proper length, and are sometimes actually paralyzed for a time by this extension, while, on the contrary, other muscles are relaxed so completely that they remain for a considerable period incapable of performing their proper functions. Yet when a few months have passed, and the elements of a new joint are constructed at the place where the extremity of the misplaced bone reposes, both these sets of muscles recover very nearly their original powers. This change of circumstances results not from changes of tonicity alone, but even more considerably from a modification of nutrition, in consequence of which the shortened muscles have their fibres rendered permanently shorter, and those which are put upon the stretch actually increase in length. This law it is which produces the chief part of the difficulty in cases of not very recent date. Were the *habitual tonic contraction* the sole opposing force, it would be very readily overcome at any time by a long continued application of a gentle, but constant extending apparatus. Now, waving all argument in relation to congenital club-foot, the consideration of the reputed causes of which would lead me into discussions for which I have neither time nor space at the present moment, let us glance at the condition of the extensor muscles in one of the most common forms of what is rather objectionably called the "acquired" varus, or pes equinus.

Suppose a youth to be affected with an intractable sore, bruise or wound, upon the heel, and to be consequently induced to tread constantly upon the anterior part of the foot or toes. The extensors of the foot are then called into play more constantly and powerfully than natural. They are developed to the greatest degree by this exercise, while their antagonists, the flexors, are actually losing strength by continued functional repose. The tonic contraction of the extensors being thus increased by habitual exertion, while the opposing forces are diminished in energy by habitual rest, the foot assumes a position of undue extension, not only while the patient is walking, but even when buried in slumber. As a necessary consequence, the muscles of the calf become habituated to the new posture, and undergo changes precisely similar to those observed in cases of luxation. To use a provincial expression, *they grow shorter* permanently by a modification of their nutrition, and cannot be again restored to their natural condition by merely overcoming their tonicity.

The same forces which thus draw and retain the limb in an unusual attitude, produce equally important changes in the form of the bones and the condition of the ligaments, as has been already stated in a previous note; and these effects render even the confirmed shortening of the extensor muscles a secondary, rather than a primary difficulty in the treatment. But the means by which the deformity is produced, are generally effective in removing it. That which causes a contraction of the extensors and a relaxation of the flexors may be made, by suitable management, to bring about a contraction of the flexors and a relaxation of the extensors: so that it would not be altogether irrational to prescribe a sore upon parts near the little toe, in order to relieve the consequences of a sore beneath the heel.

As long as the condition just described continues, no one can discover any barrier to the cure, by simple mechanical force, of so much of the deformity as is independent of the changes of form undergone by the tarsal bone, if the force be kept up with due moderation for a sufficient length of time. But other alterations of structure in the muscles soon make their appearance. The ankle joint is kept in a great degree inactive. The weight of the body is much less frequently thrown upon the affected foot, and even when the limb is employed, that weight acts at such mechanical disadvantage on the extended toes, that the muscles of the calf are called upon for much less functional exertion to support it. Gradually those muscles and their antagonists waste away, for want of exercise, as these organs usually do in complete anchylosis, or in the limbs of the Hindoo devotees who preserve one posture for many years as a religious penance. In time, the alterations of the joints and the formation of new ligaments produce complete true or false anchylosis, and the muscles then becoming absolutely useless, they are diminished to the smallest possible dimensions and rendered totally incapable of action—yet the organs are not *destroyed*. The capsular sheath of each muscle remains, and still encloses within it the cellular tissue which was the nidus of the muscle, and which still possesses the power of regenerating it under more favourable circumstances.

In most cases of club-foot of long continuance, the condition of the principal muscles of the leg approximates, more or less nearly, this simple, or, as it may be termed, elementary state. Having had many curious opportunities of verifying the character of the changes undergone by the muscles during long-continued inactivity, I feel perfectly confident, even from external examination, that the fibrous structure of these organs has become soft and yellowish in a large majority of cases; its contractile power being nearly extinct. In some instances, the paralysis is complete; and were we then to cut into the limb, we should find the muscles reduced very nearly to the condition of simple cellular tissue, with a seemingly fibrous deposite of matter, bearing quite as near a resemblance to fat as to flesh. Now, cellular tissue is very readily extensible under the action of slight mechanical forces; and that a fascia quite as resistant as the thecæ of these muscles may yield with facility to distension of mild character, is proved by the history of pregnancy and acites. There can be no question, then, that while in this altered condition, the contracted muscles in club-foot are even more capable of yielding to a great extent before the action of mechanical forces than they are when in the healthy and vigorous state. Moreover, this yielding is not the mere result of tonic relaxation—it is accompanied by a positive growth. The muscle or its nidus becomes longer by a modification of its nutrition, as do the abdominal tendons during gestation, or the skin over an increasing tumour.

In the club-foot with wasting of the limb from want of exercise we find, then, no *argument of necessity* in favor of tenotomy. How far the operation may be defensible upon the *plea of expediency*, will be considered more fully hereafter.

But the advocates of the knife inform us that mechanical extension employed with sufficient force, throws the affected muscle into spasm, and thus increases the resistance.—Spasm in almost annihilated muscles!

There are few subjects in surgery more vexing to the feelings of one who studies the science with a view to the application of truly scientific principles to the treatment of external disease, than this question of spasm. The practitioner meets with it at every turn. A luxation takes place, and the most valuable moments are lost in bleeding, fomentations, nauseats, &c., in order to prevent all danger of *spasm* when the necessary force is applied in the reduction; (I draw from the life,) as if the head of a bone misplaced were not a more powerful irritant, and more likely to produce spasm than any warrantable amount of extension *properly directed!* Who does not know that a relaxed muscle is vastly more subject to spasm from direct irritation than one which is extended! What would we say to a physician who, when called to a patient with cramp in the calf of the leg, should direct flexion of the knee and extension of the foot to relax the affected muscles while he applied his cataplasms and fomentations? Does not the patient involuntarily leap from his bed upon the instant, if able so to do, in order to put the parts as much as possible upon the stretch? Should the practitioner endeavour to act, in these cases of common occurrence, upon the principles advocated with safety to his reputation in more occult affections, the amiable elderly lady of the family, whose experience extends to three generations, would probably administer a more effective critical reprimand than any which could reach him through the columns of a medical journal!

In cases of fracture, we meet with similar notions, under circumstances rendering them still more absurd. The sharp and jagged extremities of the fractured bone are plunged into the surrounding muscles, which are soon thrown into an inflammed condition, with highly increased irritability; but the surgeon must not attempt to lighten the pressure of the spicula upon the inflamed fibres by resisting their tonic contraction—much less may he draw them entirely away from the seat of injury by reducing the fracture—for fear of *spasm!*—Truly, a little common sense may be found more available, sometimes, than the authority of great names! If spasm occur *consecutively* in club-foot, from the malposition of the limb—an accident by no means infrequent—one of the most effectual means of conquering the difficulty would be found in the much-condemned process of mechanical extension.

When the spasmodic affection is primary—that is, when spasm is *the cause*, and not *the consequence* of club-foot—then I acknowledge that the question presents itself in a very different light. In this case, the spasmodic action results not from any peculiar irritability of the muscle, but in the condition of the nerve, or nervous centre on which its motions depend. I am by no means prepared to acknowledge that extension is improper in all cases of spasm of this character occurring in club-foot; for, in common cramp, the cause of the contraction is usually an irritation seated in the small intestines, and yet it may be instantly relieved in, or, rather, driven from any particular muscle by putting it and keeping it fully upon the stretch. But the propriety of extension under such circumstances is an unsettled and very doubtful question. We know at present very little of the causes of those nervous affections which produce deformities. Very many of them are congenital, and we must quietly await the progress of transcendental anatomy, physiology, and pathology—subjects which have attracted little available attention till within the last twenty years—before we can act otherwise than empirically upon them.* But let it be granted, for the sake of argument, that extension, during the actual existence of primary or causative spasm, is interdicted in club-foot! Does it follow that tenotomy is indicated? Would any rational surgeon defend the position that the division of the tendo Achilles during the violent spasmodic contraction of the gastrocnemus and soleus muscles is calculated to lessen the difficulties of the case, and increase the chance of a restoration of the foot to a natural and useful condition? What would be the immediate consequences of the operation? It does not touch the seat of disease in any degree. That seat is in the nervous, and not in the muscular system. The only useful and direct effect of the incision is the removal of local irritation, attended, *perhaps*, with considerable pain. Among the evils resulting immediately, is the sudden con-

* I do not use the word "empirically" in an invidious sense. Much of the most solid experience and precept in modern medicine is still necessarily empirical.

traction of the muscles to their smallest possible longitudinal dimensions, and their permanent retention in that position.

The distance between the divided extremities of the tendon is then at once brought to its maximum, and a very long portion of new formed matter must be interposed between them in the process of cure. It is in vain to pretend that, in this condition, the muscles can perform their proper functions. They are reduced, together with their tendon, nearly to the effective condition of a simple ligament; and the patient may remain for life incapable of rising on the toe—a motion of the utmost importance to easy progression.

But, it will be stated by the advocates of the knife that such results are never seen in actual practice. I am well aware that if they do occur, they must be very rare, never having met with or heard of one; but I am arguing here *the hypothesis, and not the practice.* The reason why they are not seen, though they appear inevitable according to the doctrine of spasm, is simply this: the hypothesis is defective. However spasmodic the case may be in the first instance—and it is rarely of this character except in true "acquired pes equinus,"—it soon ceases to be so from the action of natural causes; and, while it continues decidedly spasmodic, the operation of tenotomy is more strongly counter-indicated than is the mechanical treatment.

Let me explan this matter more definitely. One of the first effects of the spasmodic affection is to bring the muscles of the limb to a state of absolute rest, so far as their proper functional exercise is concerned. The contraction, by the hypothesis, is permanent, and not paroxysmal, and the condition of rest is therefore equally constant. But, by an unvarying physiological law, organs kept permanently in a state of functional rest undergo a series of alterations by the modification of the nutritive process, which reduces them more and more nearly to the condition of simple cellular tissue; in which condition a muscle becomes, by the destruction of its peculiar contractility, absolutely incapable of spasm. The examination of numerous cases convinces me that, in all patients whose deformity has been of tolerably long continuance, the extensors approach to this state of simplicity of structure, and that, in the worst cases, they approximate indefinitely nearly to masses of cellular tissue, with a deposit of matter resembling yellowish fat. The continuance of active spasmodic resistance to mechanical extension, under such circumstances, is impossible. The hypothesis is a dream! Whence, then, is the source of the great pain attributed to spasm, when the mechanical treatment is vigorously applied in cases of club-foot? It can be the result of nothing else than the unwarrantable employment of too great or too sudden force, acting upon the stretched tissues of the paralysed and diminished muscles or their thecæ. The objection holds against the improper management of the surgeon, and not against the essential character of the method of treatment.

When, in recent or moderate cases, spasm actually occurs, either as a cause or an effect of the deformity, both tenotomy and mechanical extension are alike interdicted. As recent arrangements are about to place me in more immediate connection with your valuable journal, I hope to find a more suitable opportunity, during the approaching year, to dilate upon this interesting subject of muscular spasm in cases of luxation, fracture, &c. At present, this is unnecessary.

If asked how it happens that the flexors are seen to act, and even to act very availably, within a few weeks after the division of the tendo Achilles, when, by my argument, it would appear that they are changed to such a degree as to have lost their functional power, I can only reply that, in a large majority of cases, the muscular structure is not wholly destroyed,—that, in many instances, *a feeble functional power remains,*—and that, even in the worst and oldest forms of the complaint, the nidus of the muscle retains the power of re-creating the organs which it originally constructed in the fœtus. This operation of re-creating or remodelling the parts, will be more fully dwelt upon in my next.

The present article has been written currente calamo, under the pressure of very painful duties, with *the devil* at my elbow, faintly whispering "*more copy*,"—so that, while willing to be judged by its expressed opinions, you will excuse me for offering an apology for its unfinished and defective style.

